# Role of Four ABC Transporter Genes in Pharmacogenetic Susceptibility to Breast Cancer in Jordanian Patients

**DOI:** 10.1155/2019/6425708

**Published:** 2019-07-17

**Authors:** Laith N. AL-Eitan, Doaa M. Rababa'h, Mansour A. Alghamdi, Rame H. Khasawneh

**Affiliations:** ^1^Department of Applied Biological Sciences, Jordan University of Science and Technology, Irbid 22110, Jordan; ^2^Department of Biotechnology and Genetic Engineering, Jordan University of Science and Technology, Irbid 22110, Jordan; ^3^College of Medicine, King Khalid University, Abha, Saudi Arabia; ^4^Department of Hematopathology, King Hussein Medical Center (KHMC), Jordanian Royal Medical Services (RMS), Amman 11118, Jordan

## Abstract

Breast cancer pharmacogenetics is increasingly being explored due to chemotherapy resistance among certain classes of patients. The ATP binding cassette (ABC) transporter genes have been previously implicated in breast cancer progression and drug response. In the present study, single nucleotide polymorphisms (SNPs) from the* ABCC1*,* ABCC2*,* ABCB1*, and* ABCG2* genes were screened in breast cancer patients and healthy volunteers from the Jordanian-Arab population. Only the* ABCB1 *SNPs showed a significant association with BC in Jordanian-Arab patients, and the* ABCB1* SNP rs2032582 exhibited a strong genotypic association with BC. With regard to the clinical characteristics of BC, the* ABCC2 *SNPs rs2273697 and rs717620 were found to be significantly associated with age at breast cancer diagnosis and breastfeeding status, while the* ABCB1* SNP rs1045642 was significantly associated with age at breast cancer diagnosis. In terms of pathological characteristics, the* ABCC1 *SNP rs35628 and the* ABCB1* SNP rs2032582 were significantly associated with tumor size, the* ABCC2 *SNP rs2273697 was significantly associated with estrogen receptor status, and the* ABCG2 *SNP rs2231142 was significantly associated with axillary lymph node status. In this current study, we assume that significant genetic variants within the ABC superfamily may increase the risk of breast cancer among Jordanian women. Furthermore, these variants might be responsible for worse BC prognosis.

## 1. Introduction

Breast cancer (BC) is the most common female malignancy in the majority of countries [1]. Arab populations suffer from lower but steadily rising BC incidence rates compared to their American and European counterparts, and the clinical characteristics of the disease also differ between the aforementioned populations [2]. Such population-level differences in BC predisposition have been attributed to genetics and have been widely investigated, with different mutations having different levels of association with BC [3]. Compounding this issue is the fact that Arab BC genetics are not well researched, and much less is known about the genes involved in BC progression and drug response in Arab patients [4].

The ATP binding cassette (ABC) transporters comprise seven subfamilies of membrane proteins that facilitate the transport and modulate the effects of a wide range of drugs and their metabolites [5, 6]. Remarkably, an overexpression of certain ABC transporters in cancer cell lines resulted in multidrug resistance (MDR) and a potential failure of chemotherapy [7, 8]. For example, the* ABCC1* gene, also known as multidrug resistance-associated protein 1 (*MRP1*), is associated with worsened prognoses in a wide range of tumors, while the* ABCC2* gene was found to contribute to drug resistance [9, 10]. Likewise, the* ABCB1 *gene is highly polymorphic and induces chemoresistance by preventing drug accumulation in cancer cells [7]. In addition, the* ABCG2* gene, also known as the breast cancer resistance protein (*BCRP*), is responsible for the transport of many conventional chemotherapeutics and causes MDR in various cancer cells [11].

In the present study, four SNPs of ABC transporter genes, namely* ABCC1*,* ABCC2*,* ABCB1*, and* ABCG2*, were screened in Jordanian Arabs with and without breast cancer. Previous reports have indicated that these genes play a critical role in increasing tumor risk, especially in breast cancer [9, 11]. The aim of this study is to determine whether the aforementioned genes play a significant role in Jordanian breast cancer patients.

## 2. Materials and Methods

### 2.1. Ethical Approval and Conduct

The present study was given ethical approval by the Institutional Review Board (IRB) at Jordan University of Science and Technology. Written informed consent was obtained from all participants in this study before blood sample withdrawal.

### 2.2. Study Population and Design

The study cohort consisted of 222 women diagnosed with breast cancer as well as 218 healthy matched volunteers. All participants were recruited from the Jordanian population and were of Arab descent. 5 ml of blood were withdrawn from each participant into EDTA tubes and refrigerated until DNA extraction.

### 2.3. Genomic Extraction and Genotyping

Genomic DNA was extracted from a total of 440 blood samples using the Wizard® Genomic DNA Purification Kit (Promega, USA). Extracted DNA was evaluated in terms of concentration (ng/*μ*l) and purity (A260/280) quantity using the Nano-Drop ND-1000 UV-Vis Spectrophotometer (BioDrop, UK). DNA samples were then loaded onto an agarose gel to confirm product quality. Samples that met our requirements were diluted using nuclease-free water for a final concentration of 20 ng/*μ*l and a final volume of 30 *μ*l. Genotyping was carried out by the Melbourne node of the Australian Genome Research Facility (AGRF) using the Sequenom MassARRAY® system (iPLEX GOLD) (Sequenom, San Diego, CA, USA).

### 2.4. Denomination of Genotypic-Phenotypic Correlation

In this study, several clinical and pathological features of BC were investigated in correlation with the studied variants. Clinical and pathological information for patients was collected from their medical records. P values were selected to estimate the association between SNPs and risk of BC. The analyses were done per genotype.

### 2.5. Statistical Analysis

Case-control analyses were carried out using different statistical software. Allelic and genotypic frequencies were calculated using the Hardy-Weinberg equilibrium (HWE) equation (Court lab - HW calculator) (http://www.oege.org/software/hwe-mr-calc.html). The Statistical Package for the Social Sciences (SPSS), version 25.0 (SPSS, Inc., Chicago, IL) was used to calculate the p values that allowed discrimination between cases and controls in association with the genotype. It also facilitated the analysis of the different genotype models. On the other hand, genotype-phenotype assessment was performed using the Chi-Square test and ANOVA tests [12]. P value denoted statistical significance if they were less than 0.05.

## 3. Results

### 3.1. ABC Transporter Variants and Their Minor Allele Frequencies (MAF)

Table 1 displays the SNPs of the* ABCC1*,* ABCC2*,* ABCB1*, and* ABCG2* candidate genes. All of the polymorphic SNPs were tested for minor allele frequencies (MAF) and HWE p values in both the cases and controls (Table 1).

### 3.2. Association between ABC Transporter SNPs and Breast Cancer (BC)

**Figure 1:**
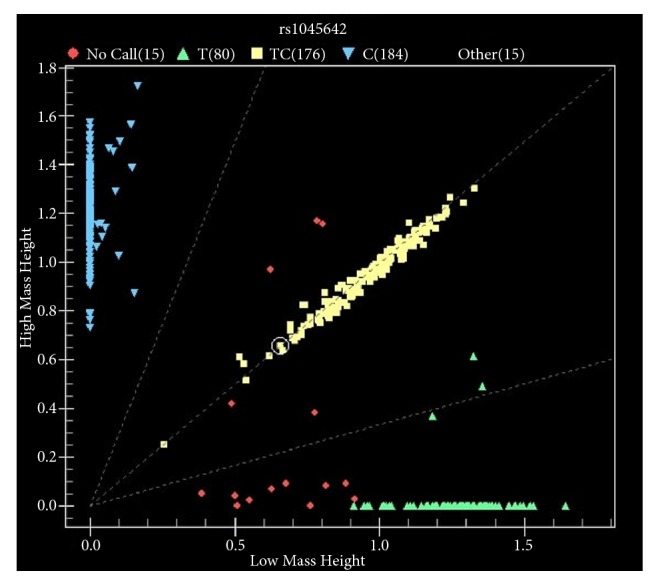
Scatter plot for rs1045642 within* ABCB1* gene. Each Dot represents a sample while different genotypes are indicated with different colors.

The allelic and genotypic frequencies of the ABC transporter SNPs were determined for both cases and controls (Table 2). All three* ABCB1* SNPs were found to be significantly associated with BC in Jordanian patients, with rs1045642, rs1128503, and rs2032582 having p values of 0.01164587, 0.01610842, and 0.03565022, respectively.  Figure 1 shows a representative scatter pattern for rs1045642 of* ABCB1*. In contrast, only the rs2032582 SNP of* ABCB1* showed a strong genotypic association with BC (p value = 1e^-8,^ OR =6.72, 95% CI = 4.27 to 10.57). rs2032582 is a triallelic polymorphism comprising the A, C, and T (minor) alleles (the homozygous TT variant was not estimated in the current study population). None of the other investigated SNPs showed any significant correlation with BC, as all the allelic and genotypic frequencies were greater than 0.05 (Table 2).

Further genetic analyses were carried out to test for the association of different genetic models with BC.  Table 3 summarizes three different genetic models and the chi-squared value for each. The* ABCG2* gene was excluded from the analysis because it expressed only two genotypes. For the* ABCC1 *SNP rs4148351, Het (CT) versus Common Hz (CC) was found to be associated with BC in Jordanian Arabs (*χ*2 = 5.33; p value <0.05). Similarly, for the* ABCB1* SNP rs1128503, the Rare Hz (AA) versus Common Hz (GG) model was related to BC in Jordanian Arabs (*χ*2 =4.52; p value <0.05). No such association was found for any of the* ABCC2* SNPs (Table 3).

### 3.3. Association between ABC Transporter SNPs and Major Prognostic Factors of Breast Cancer (BC)

Certain clinical and pathological characteristics of BC serve as major prognostic factors for the disease that are exploited in the process of treatment selection. None of the* ABCC1 *SNPs showed any significant association with the clinical characteristics of BC, but the* ABCC2 *SNPs rs2273697 and rs717620 were found to be significantly associated with age at breast cancer diagnosis (p value = 0.042) and breastfeeding status (p value = 0.05), respectively (Table 4). Meanwhile, the* ABCC1 *SNP rs35628 was associated with the pathological characteristic of tumor size (p value = 0.014), while the* ABCC2 *SNP rs2273697 was significantly associated with estrogen receptor status (p value = 0.013) (Table 4).

Likewise, rs1045642 was the only* ABCB1* SNP to be significantly associated with a clinical characteristic of BC, namely, age at breast cancer diagnosis (p value = 0.029) (Table 5). In contrast, rs2032582 was the only* ABCB1* SNP to show significant association with a pathological characteristic of BC, namely, tumor size (p value = 0.03) (Table 5). The* ABCG2 *SNP rs2231142 was found to be significantly associated with axillary lymph node status (p value = 0.001) but not with any clinical characteristic (Table 5).

### 3.4. Association between ABC Transporter SNPs and Immunohistochemistry (IHC) Profiles of Breast Cancer (BC)

Different combinations of the progesterone receptor, estrogen receptor, and Her2/neu expression molecular markers gives rise to three different immunohistochemistry profiles: Luminal A, Luminal B, and Triple Negative. These profiles and their correlation with the investigated SNPs are displayed in Tables 4 and 5. Only the* ABCC1 *SNP rs35626 was found to be significantly correlated with the different IHC profiles (p value = 0.013).

## 4. Discussion

In the present study, four ABC transporter genes were screened in female BC patients and healthy volunteers from Jordan. Three SNPs from each of the* ABCC1, ABCC2, *and* ABCB1 *genes and one SNP from the* ABCG2* gene were investigated for their association with BC in patients of Jordanian-Arab descent.

The* ABCC1 (MRP1) *gene has been previously reported as being a predictor of hematological toxicity in BC patients undergoing certain chemotherapy regimens [13]. It has also been found to be involved in MDR development in cases of neuroblastoma [14]. Moreover,* ABCC1* expression was found to be increased in children with acute lymphoblastic leukemia, and* ABCC1 *gene induction resulted in worsened disease-free and overall survival rates [15, 16]. Our results show that none of the three investigated* ABCC1* SNPs showed any significant association with the clinical and pathological characteristics of BC. However, we found that the* ABCC1 *SNP rs35626 was significantly associated with different immunohistochemistry (IHC) profiles in Jordanian-Arab patients.

Similar to* ABCC1*, the* ABCC2 *gene is involved in decreased recurrence-free survival in BC patients receiving tamoxifen [17]. Nuclear expression of* ABCC2* in BC cells was also found to be associated with worsened clinical outcome [18]. Our findings showed that the* ABCC2 *SNP rs2273697 was significantly associated with age at breast cancer diagnosis. Furthermore, rs2273697 was in correlation with estrogen receptor status for genotype association, patients were categorized according to the expression of estrogen receptor (positive versus negative) and tested with regard to their genotypes. However, in this study only gender was matched for the analysis. In addition, rs717620 was associated with breastfeeding status.

Three* ABCB1* SNPs rs1045642, rs1128503, and rs2032582 have been suggested to play a role in altered doxorubicin pharmacokinetics in Asian BC patients [19]. In the present study, all three aforementioned* ABCB1 *SNPs were significantly associated with BC in Jordanian Arabs. Moreover, the* ABCB1* SNPs rs1045642 and rs2032582 were significantly associated with age at breast cancer diagnosis and tumor size, respectively.

Overexpression of the* ABCG2 *gene was implicated in developing flavopiridol resistance in BC cells [20]. The homozygous genotype (CC) of the* ABCG2* SNP rs2231142 of the* ABCG2 *gene resulted in significantly reduced intestinal transport activity compared to the wildtype (AA) [21]. In Kurdish BC patients, the A allele of the rs2231142 SNP may be a risk factor for BC progression, while the C allele was associated with poorer responses to anthracyclines and paclitaxel [22]. In contrast, the homozygous (CC) genotype of the* ABCG2* SNP rs2231142 was significantly associated with longer progression-free survival in Han Chinese BC patients [23]. In the present study, the* ABCG2 *SNP rs2231142 was found to be significantly associated with axillary lymph node status in Jordanian BC patients.

Conclusively, screening certain ABC transporter genes in BC patients and healthy volunteers from the Jordanian-Arab population revealed a number of interesting observations. Perhaps the most important finding was that the* ABCB1 *SNPs were the only variants to be significantly associated with BC in Jordanian Arabs.

## Figures and Tables

**Table 1:** Minor allele frequencies among breast cancer patients and healthy controls and the HWEc p value of ABC gene polymorphisms.

Gene	SNP ID	SNP position ^a^	Cases (n = 222)				Controls (n = 218)	
			MA^b^	MAF^c^	HWE^d^	MA^b^	MAF^c^	HWE^d^
								p-value
					p-value			
ABCC1	rs35626	16076758	T	0.38	0.3	T	0.41	0.12
	rs35628	16077249	G	0.1	0.049	G	0.11	0.27
	rs4148351	16076711	T	0.16	0.037	T	0.2	N/A

ABCC2	rs2273697	99804058	A	0.25	N/A	A	0.24	0.089
	rs3740065	99845936	G	0.23	N/A	G	0.21	0.066
	rs717620	99782821	T	0.12	0.75	T	0.13	0.38

ABCB1	rs1045642	87509329	T	0.35	0.025	T	0.43	0.026
	rs1128503	87550285	A	0.36	0.039	A	0.44	0.074
	rs2032582	87531302	T	0.03	0.4591	T	0.01	0.615

ABCG2	rs2231142	88131171	T	0.04	0.552	T	0.04	0.572

^a^Chromosome positions are based on NCBI Human Genome Assembly Build. ^b^MA: minor allele. ^c^MAF: minor allele frequency. ^d^HWE: Hardy–Weinberg equilibrium. N/A: not applicable.

**Table 2:** Association of the investigated ABCC1, ABCC2, ABCB1, and ABCG2 SNPs and breast cancer (BC).

Gene	SNP ID	Allelic and Genotypic Frequencies in Cases and Controls				
		Allele/Genotype	Cases	Controls	P-value*∗*	Chi-square
			(n = 222)	(n = 218)		
*ABCC1*	rs35626	G	283(0.65)	256 (0.59)	0.073	3.214
		T	155 (0.35)	180 (0.41)		
		GG	95 (0.43)	81(0.37)	0.216	3.063
		GT	93 (0.42)	94(0.43)		
		TT	31 (0.14)	43 (0.2)		
	rs35628	A	394 (0.9)	388 (0.89)	0.788	0.072
		G	44 (0.1)	46 (0.11)		
		AA	180(0.82)	175(0.81)	0.820	0.395
		AG	34(0.16)	38(0.18)		
		GG	5(0.02)	4(0.02)		
	rs4148351	C	369(0.84)	346(0.8)	0.082	3.021
		T	69 (0.16)	88 (0.2)		
		CC	160 (0.73)	138 (0.64)	0.068	5.374
		CT	49 (0.22)	70(0.32)		
		TT	10 (0.05)	9(0.04)		

*ABCC2*	rs2273697	G	332(0.75)	331(0.76)	0.778	0.079
		A	108 (0.25)	103 (0.24)		
		AA	13 (0.06)	17(0.08)	0.412	1.773
		GA	82 (0.37)	69(0.32)		
		GG	125 (0.57)	131(0.6)		
	rs3740065	A	341(0.77)	345(0.79)	0.478	0.503
		G	101(0.23)	91(0.21)		
		AA	131(0.59)	141(0.65)	0.285	2.51
		AG	79(0.36)	63(0.29)		
		GG	11(0.05	14(0.06)		
	s717620	C	387(0.88)	377(0.87)	0.285	2.51
		T	55(0.12)	57 (0.13)		
		CC	170(0.77)	165(0.76)	0.928	0.149
		CT	47(0.21)	47(0.22)		
		TT	4(0.02)	5(0.02)		

*ABCB1*	rs1045642	C	288(0.65)	248(0.57)	0.012	6.364
		T	152(0.35)	186(0.43)		
		CC	102(0.46)	79(0.36)	0.063	5.499
		CT	84(0.38)	90(0.41)		
		TT	34(0.15)	48(0.22)		
	rs1128503	A	278(0.64)	242(0.56)	*0.016*	5.791
		G	158(0.36)	192(0.44)		
		AA	36(0.17)	49(0.23)	0.074	5.189
		GA	86(0.39)	94(0.43)		
		GG	96(0.44)	74(0.34)		
	rs2032582	A	144(0.33)	174(0.43)	*0.035*	6.668
		C	284(0.65)	252(0.58)		
		T	12(0.03)	6(0.01)		
		AA	29(0.13)	41(0.19)	*1e-8*	44.386
		CA	82(0.37)	90(0.42)		
		CC	97(0.44)	79(0.37)		
		TA	49(0.02)	2(0.0093)		
		TC	8(0.04)	4(0.02)		

*ABCG2*	rs2231142	T	17(0.04)	16(0.04)	0.902	0.015
		G	425 (0.96)	418 (0.96)		
		GG	204(0.92)	201(0.93)	0.899	0.016
		GT	17(0.08)	16(0.07)		

P value <0.05 was considered as significant.

**Table 3:** Genetic association analysis for the ABCC1, ABCC2, ABCB1, and ABCG2 SNPs using different genetic models.

Gene	SNP ID	Category Test	Odds Ratio	95% CI	Chi square*∗*
*ABCC1*	rs35626	Het (GT) vs. Common Hz (GG)	0.84	0.56-1.27	0.65
		Rare Hz (TT) vs. Het (GT)	0.73	0.42-1.25	1.31
		Rare Hz (TT) vs. Common Hz (GG)	0.61	0.36-1.06	3.04
	rs35628	Het (AG) vs. Common Hz (AA)	0.87	0.52-1.44	0.29
		Rare Hz (GG) vs. Het (AG)	1.4	0.35-5.63	0.22
		Rare Hz (GG) vs. Common Hz (AA)	1.22	0.32-4.6	0.08
	rs4148351	Het (CT) vs. Common Hz (CC)	0.6	0.39-0.93	5.33
		Rare Hz (TT) vs. Het (AG)	1.58	0.6-4.19	0.88
		Rare Hz (TT) vs. Common Hz (CC)	0.96	0.38-2.43	0.01

*ABCC2*	rs2273697	Het (GA) vs. Common Hz (GG)	1.55	0.71-3.42	1.21
		Rare Hz (AA) vs. Het (GA)	0.8	0.54-1.2	1.14
		Rare Hz (AA) vs. Common Hz (GG)	1.25	0.58-2.68	0.32
	rs3740065	Het (GA) vs. Common Hz (AA)	1.35	0.9-2.03	2.08
		Rare Hz (GG) vs. Het (GA)	0.63	0.27-1.48	1.16
		Rare Hz (GG) vs. Common Hz (AA)	0.85	0.37-1.93	0.16
	rs717620	Het (CT) vs. Common Hz (CC)	0.97	0.61-1.53	0.02
		Rare Hz (TT) vs. Het (CT)	0.8	0.2-3.17	0.1
		Rare Hz (TT) vs. Common Hz (CC)	0.78	0.2-2.94	0.14

*ABCB1*	rs1045642	Het (CT) vs. Common Hz (CC)	0.72	0.48-1.1	2.32
		Rare Hz (TT) vs. Het (CT)	0.85	0.5-1.43	0.39
		Rare Hz (TT) vs. Common Hz (CC)	0.61	0.37-1.03	3.46
	rs1128503	Het (GA) vs. Common Hz (AA)	1.25	0.74-2.1	0.68
		Rare Hz (GG) vs. Het (GA)	1.42	0.93-2.16	2.65
		Rare Hz (GG) vs. Common Hz (AA)	1.77	1.04-2.99	4.52

*∗* For significant association *χ*2 should be >3.84 with P<0.025.

CI indicates confidence interval.

**Table 4:** Association between different ABCC1 and ABCC2 SNP genotypes and the clinicopathological characteristics of breast cancer (BC).

Clinical characteristics	*ABCC1*			*ABCC2*		
	rs35626 GG vs GT vs TT	rs35628 AA vs AG vs GG	rs4148351 CC vs CT vs TT	rs2273697 AA vs AG vs GG	rs3740065 AA vs AG vs GG	rs717620 CC vs CT vs TT
Body mass index *∗∗*	0.535	0.116	0.068	0.813	0.461	0.084

Age at first pregnancy *∗∗*	0.990	0.624	0.358	0.381	0.921	0.458

Age at BC diagnosis *∗∗*	0.311	0.352	0.198	0.042	0.194	0.104

Allergy *∗*	0.808	0.824	0.867	0.501	0.324	0.065

Age at menarche *∗∗*	0.219	0.824	0.373	0.820	0.747	0.611

Breastfeeding status *∗*	0.284	0.117	0.761	0.439	0.340	0.005

Age at menopause *∗∗*	0.437	0.665	0.373	0.115	0.155	0.251

Family history *∗*	0.669	0.605	0.762	0.472	0.891	0.415

Comorbidity *∗*	0.764	0.967	0.976	0.130	0.741	0.140

Smoking *∗*	0.237	0.287	0.163	0.320	0.406	0.362

Pathological characteristics						

Progesterone receptor status *∗*	0.292	0.516	0.244	0.610	0.823	0.423

Estrogen receptor status *∗*	0.730	0.550	0.562	0.013	0.839	0.125

HER2 *∗*	0.146	0.500	0.330	0.441	0.226	0.842

IHC profile*∗*	0.013	0.838	0.260	0.381	0.775	0.270

Tumor differentiation *∗*	0.754	0.940	0.963	0.768	0.718	0.431

Axillary lymph nodes *∗*	0.113	0.184	0.817	0.138	0.989	0.213

Tumor stage *∗*	0.491	0.751	0.665	0.748	0.999	0.357

Histology classification *∗*	0.963	0.502	0.348	0.301	0.294	0.661

Tumor size *∗∗*	0.888	0.014	0.968	0.720	0.576	0.922

Lymph node involvement *∗*	0.694	0.944	0.794	0.165	0.339	0.528

*∗* Pearson's chi-squared test was used to determine genotype-phenotype association.

*∗∗* Analysis of variance (ANOVA) test was used to determine genotype-phenotype association.

P value <0.05 was considered as significant.

**Table 5:** Association between different ABCB1 and ABCG2 SNP genotypes and the clinicopathological characteristics of breast cancer (BC).

Clinical characteristics	*ABCB1*			*ABCG2*
	rs2032582 A vs C vs T	rs1128503 AA vs AG vs GG	rs1045642 CC vs CT vs TT	rs2231142 GG vs GT
Body mass index *∗∗*	0.298	0.383	0.180	0.164

Age at first pregnancy *∗∗*	0.212	0.326	0.815	0.490

Age at BC diagnosis *∗∗*	0.931	0.924	*0.029*	0.592

Allergy *∗*	0.310*∗*	0.331	0.169	0.511

Age at menarche *∗∗*	0.508	0.525	0.115	0.947

Breastfeeding status *∗*	0.708	0.291	0.665	0.553

Age at menopause *∗∗*	0.746	0.258	0.676	0.563

Family history *∗*	0.585	0.626	0.469	0.481

Comorbidity *∗*	0.350	0.347	0.751	0.341

Smoking *∗*	0.462	0.365	.303	0.429

Pathological characteristics				

Progesterone receptor status *∗*	0.375	0.555	0.268	0.244

Estrogen receptor status *∗*	0.470	0.480	0.299	0.312

HER2 *∗*	0.712	0.886	0.835	0.560

IHC profile*∗*	0.186	0.645	0.160	0.606

Tumor differentiation *∗*	0.429	0.632	0.595	0.926

Axillary lymph nodes *∗*	0..373	0.718	0.847	0.001

Tumor stage *∗*	0.700	0.705	0.723	0.722

Histology classification *∗*	0.488	0.498	0.602	0.648

Tumor size *∗∗*	0.030	0.032	0.556	0.249

Lymph node involvement *∗*	0.021	0..056	0.417	0.381

*∗* Pearson's chi-squared test was used to determine genotype-phenotype association.

*∗∗* Analysis of variance (ANOVA) test was used to determine genotype-phenotype association.

P value <0.05 was considered as significant.

## Data Availability

The datasets generated and/or analysed over the course of the study are not publicly available but are available from the corresponding author upon reasonable request.
